# Drug prescription pattern in exotic pet and wildlife animal practice: a retrospective study in a Spanish veterinary teaching hospital from 2018 to 2022

**DOI:** 10.3389/fvets.2023.1328698

**Published:** 2024-01-08

**Authors:** Beatriz Romero, Julen Susperregui, Ana M. Sahagún, Nélida Fernández, Cristina López, Raúl de la Puente, José R. Altónaga, Raquel Díez

**Affiliations:** ^1^Department of Biomedical Sciences, Veterinary Faculty, Institute of Biomedicine (IBIOMED), University of León, León, Spain; ^2^Applied Mathematics, Department of Mathematics, University of León, León, Spain; ^3^Department of Veterinary Medicine, Surgery and Anatomy, Director of the Veterinary Teaching Hospital of the University of León (HVULE), University of León, León, Spain

**Keywords:** antibiotic, prescription pattern, exotic animal, veterinary teaching hospital, wildlife animal

## Abstract

Exotic companion animals have had an important role in our society since ancient times. Preserving animal health is necessary to do a responsible use of veterinary medicines. This study aimed to describe the prescription patterns of drugs in exotic pets and wildlife animals attending the Veterinary Teaching Hospital of the University of León (HVULE). A retrospective study was carried out between 2018 and 2022. Birds were the largest group of exotic animals attending the HVULE. Visits were related to emergency reasons and for musculoskeletal disorders. One-third of the animals were eventually euthanised. Regarding pharmacological treatments, the most frequently active ingredients used were pentobarbital, isoflurane, meloxicam, and within antibiotics, marbofloxacin (category B in the classification of European Medicines Agency).

## 1 Introduction

The adjective *exotic* is a term with several definitions, all equally valid to define an *exotic animal*. However, there are other expressions more commonly used, such as non-traditional or non-domestic animals (1). The American Veterinary Medical Association (AVMA) considers *exotic companion animals* as all species that coexist with humans, both domestic and non-domestic, representing hundreds of animal species of reptiles, amphibians, birds and mammals, the Pisces group, and the phylum *Arthropoda* (2). In Spain, Royal Decree 630/2013, which regulates the Spanish catalog of invasive exotic species, defines an *exotic pet animal* as “an animal of non-native wildlife that is individually dependent on humans, lives with them and has assumed the habit of captivity” (3).

Although according to the AVMA, dogs and cats are by far the largest companion animal population in the United States, between 2017 and 2018, the ownership of other types of pets, such as fish, ferrets, rabbits, hamsters, guinea pigs, turtles, snakes, and lizards, has increased significantly (4). In Europe, the number of ornamental birds is estimated to be approximately 50 million, whereas ornamental fish are approximately 300 million, small mammals 27 million, and reptiles 8 million (5). In Spain, according to data from 2021, there are more than 29 million pets, including nearly 8 million fish, 5 million birds, 1.5 million reptiles, and 1.5 million small mammals (6). However, the recent Law 7/2023 on the Protection of the Rights and Welfare of Animals may change this trend in this country, as it prohibits “the keeping, breeding, trade, sale, offer for sale, exchange or donation and import or export as pets of individuals of species not included in a positive list of pets” (7), which has not yet established.

This growing demand for exotic animals poses new challenges for veterinarians, as they are expected to effectively care for, diagnose, and treat a huge variety of animal species. Specifically, in the case of the different groups of exotic animals, both companion and wild ones, their anatomy and physiology may vary enormously, and also the way in which disorders manifest themselves. This makes the selection and prescription of medicines for these patients a complex process, as pharmacokinetic, efficacy, and safety data are often lacking in exotic animal medicine (8). In many cases, this often leads to the use of drugs in an extralabel manner (9) and, consequently, to an increased risk of adverse effects.

In addition, exotic practitioners are aware that the prevalence of daily emergency events, particularly those resulting in death, is higher among exotic animals than with other species such as dogs or cats (2). Among the reasons that may explain this fact is that most exotic species, as prey animals, tend to mask their diseases. Furthermore, the interaction time of owners with these animals may not be as long as with a dog or cat. Moreover, the behavioral patterns indicative of pain or disease in these animal species are not well known, which may lead to treatment failure to provide help to the animal when it is essential (2).

Among global health problems, zoonosis and antimicrobial resistance best illustrate the *One Health* concept (10). This approach is a global strategy that recognizes the interdependence among human, animal, and environmental health (11, 12), and they should be considered as a whole (13), as animal health is fundamental to ensuring public health, food security and supply, the economy, and preservation of animal species (14). Moreover, these species may be a major source of zoonoses, especially in young children and immunocompromised people (15). As for antimicrobial resistance, several studies have pointed out that antimicrobials are not often used in companion animals according to European recommendations (16–21), thus favoring the development of resistance (22, 23). Recently, increasing attention has been paid to the role of companion animals as potential reservoirs of resistant and multi-resistant pathogens for humans (24–27) as we live in close contact with them (24–26). However, in exotic animal medicine little or no information is available on antimicrobial resistance. A study carried out in wildlife rescue centers revealed that birds may be housed for long periods of time and receive antimicrobial treatments, which could potentially lead to the acquisition and subsequent colonization by antimicrobial-resistant bacteria (28).

In veterinary medicine, drugs are available to preserve animal health provided they are used responsibly (14). Rational use of medicines is based on using the right drug, at the right dose and at the right cost, as described by World Health Organization (WHO) (29). In humans, the assessment of drug utilization patterns with WHO indicators is increasingly necessary to promote rational use of medicines (30, 31). In veterinary practice, these indicators are not still defined, although antimicrobials are the drugs for which most progress has been made. In the European Union (EU), Regulation 2019/6 states that within 8 years from 28 January 2022, data on antimicrobial medicinal products should be collected from animals other than food-producing ones not later than in 2029 (32). More recently, Spanish regulations have had set a deadline to start the collection of pet data on 31 January 2025 (RD 666/2023) (33). As for exotic animals, information is scarce and scattered, often only as guidelines to manage a certain animal species, disorder, or pharmacological group (9, 34, 35), or related to common practice of veterinarians with a certain type of animals (8, 36) but not about the current consumption of drugs. Therefore, this study aimed to describe the prescription patterns of medicines in exotic pets and wildlife animals attending the Veterinary Teaching Hospital of the University of León (HVULE).

## 2 Materials and methods

### 2.1 Study site

The HVULE is the Veterinary Teaching Hospital of the Faculty of Veterinary at the University of Leon (Spain). The hospital is organized into two separate clinical departments according to the target species: small animals and large ones, both with their emergency facilities. In 2023, all the teaching staff were qualified veterinarians.

### 2.2 Study design and data collection

A retrospective study was designed to evaluate the drug prescription patterns in exotic pets and wildlife patients treated at the HVULE between 1 January 2018 to 31 December 2022.

Data of all exotic pets and wildlife (birds, mammals, and reptiles, both wild and kept animals) of the 5-year period were collected through an inductive method. All data used in this study were obtained by reviewing the individual medical records stored in GestorVet (GestorVet, Las Palmas de Gran Canaria, Spain), an online management software, one of the most widely used in veterinary clinics and hospitals in Spain. For each animal, the following data were collected: history number, year of clinical record, medical check-up, species, weight, anatomical system/organ involved, if hospitalization occurred and days of hospitalization, number of treatments and if euthanasia was applied, as well as the different treatments prescribed in the HVULE, route of administration, and dosage regimen. All medications were categorized according to the WHO anatomical-therapeutic-chemical veterinary (ATCvet) classification system (37). Any combination medicine (multicomponent products) was considered as a single medicine. The European Medicines Agency (EMA) Categorization of antibiotics for use in animals was used to classify antibiotics (Category A—Avoid; Category B—Restrict; Category C—Caution; Category D—Prudence) (38). Data were exported to Microsoft Access (Microsoft Office 2019). Patients whose medical records lacked essential information, those who died before or during consultation without having received treatment and those who did not receive any treatment were excluded.

The Strengthening the Reporting of Observational Studies in Epidemiology—Veterinary Extension (STROBE-Vet) Statement was used to report data (39).

### 2.3 Calculation of the administered daily amount

The administered daily amount (in grams) was also calculated for each given drug administration. For this purpose, the following formula was used:


$$\begin{array}{l} \mathrm{Administered}\;\mathrm{Daily}\;\mathrm{Amount}\;\left(ADA\right)=\mathrm{Amount}\;\mathrm{of}\;\mathrm{drug}\\ \;\times \mathrm{Proportion}\;\mathrm{of}\;\mathrm{active}\;\mathrm{ingredient}\;\mathrm{in}\;\mathrm{this}\;\mathrm{drug} \end{array}$$

### 2.4 Calculation of the prescription diversity

Prescription diversity (PD) is defined as “the frequency and variety with which a practice prescribes pharmaceutical classes (PC) within a determined pharmaceutical family (PF)” (40). It is calculated as follows, adjusted to a 0–1 scale where 1 represents maximal diversity:


$$\mathrm{Prescription}\;\mathrm{Diversity}\;\left(PD\right)=1-\frac{\sum np\;\left(np-1\right)}{NP\;\left(NP-1\right)}$$

np is the number of prescriptions of a particular PC within a PF, and NP is the total number of prescriptions within a PF.

### 2.5 Data management and statistical analysis

Data analysis was performed with the statistical package IBM SPSS Statistics 26 (IBM Corporation, Armonk, NY, United States). Descriptive statistics (frequencies, mean, standard deviation, ranges, and percentages with 95% confidence intervals) were used to analyse the study population. Odds ratio (OR) was calculated with their respective 95% confidence intervals (95% CI). Multivariable forward-step ordinal logistic regression analysis was conducted to assess the impact of each predictor on euthanasia and antibiotic prescriptions. A *p*-value of ≤ 0.05 was always considered as significant.

## 3 Results

Data were available from a total of 17,483 medical records registered in GestorVet for the 5-year period studied. Of those, 1,420 (8.1%) were exotic pets and wildlife animals. After applying exclusion criteria, 503 animals were included in the study, which received 1,081 medical treatments.

Table 1 summarizes the results of some background information about the animals assessed. Birds were the most frequently treated, being 247 (57.8%) birds of prey or raptors (order *Falconiformes, Accipitriformes,* and *Strigiformes*) and 180 (42.2%) synanthropic birds (order *Ciconiiformes*, *Columbiformes,* and *Apodiformes*). As for mammals, 32 were rodents (45.1%), 17 lagomorphs (23.9%), 15 mustelids (21.2%), 4 hedgehogs (5.6%), and 3 other animals (4.2%). Finally, all reptiles were chelonians (5 animals, 1%). Birds were 22 times more likely to have visited the hospital for an emergency than mammals (OR = 22.1; 95% CI: 12.1–40.6). The proportion of cases evaluated increased progressively over the study period approximately 195%, despite the COVID-19 pandemic. In addition, if an animal was admitted for emergency care, it was almost 4 times more probably to be hospitalized (OR = 3.8; 95% CI: 2.3–6.1), and approximately 2 times more likely to be euthanised (OR = 1.9; 95% CI: 1.2–3.2).

**Table 1:** Background information of the exotic pets and wildlife animals visiting the HVULE (Spain) from 2018 to 2022.

Characteristics	Frequency (%) (*n* = 503)	95% CI
**Year of clinical record**		
2018	43 (8.5)	0.061–0.110
2019	57 (11.3)	0.086–0.141
2020	131 (26.0)	0.222–0.299
2021	145 (28.8)	0.249–0.328
2022	127 (25.2)	0.215–0.290
**Hospital visit**		
Emergency	400 (79.5)	0.760–0.830
Medical check-up	97 (19.3)	0.158–0.227
Hospital discharge	3 (0.6)	0.000–0.013
Surgery	2 (0.4)	0.000–0.009
Image diagnosis	1 (0.2)	0.000–0.006
**Animal class**		
Birds	427 (84.9)	0.818–0.880
Mammals	71 (14.1)	0.111–0.172
Reptiles	5 (1.0)	0.001–0.019

CI, Confidence interval.

Table 2 shows the clinical characteristics of the animals attending the hospital. For those who were hospitalized, the length of stay was 4.56 ± 6.96 days (range 1–71, median 2.0), and the mean administered treatments were 2.15 ± 1.02 (range 1–7, median 2.0). Birds were again 3.4 times more probably to be hospitalized than mammals (OR = 3.4; 95% CI: 1.7–5.9) and 8.4 times more likely to be euthanised (OR = 8.4; 95% CI: 3.3–21.3). However, all animals undergoing surgery were mammals.

**Table 2:** Clinical characteristics of the exotic pets and wildlife animals visiting the HVULE (Spain) from 2018 to 2022.

Clinical characteristics	Frequency (%) (*n* = 503)	95% CI
**Anatomical system/organ affected**		
Musculoskeletal	297 (59.0)	0.547–0.633
Digestive	112 (22.3)	0.186–0.259
Sense organs	22 (4.4)	0.026–0.062
Respiratory	18 (3.6)	0.020–0.052
Nervous	13 (2.6)	0.012–0.040
Genitourinary	8 (1.6)	0.005–0.027
Infectious	7 (1.4)	0.004–0.024
Cardiovascular	2 (0.4)	0.000–0.009
Others*	24 (4.8)	0.029–0.066
**Euthanasia**		
No	332 (66.0)	0.619–0.701
Yes	171 (34.0)	0.299–0.381
**Hospitalization**		
No	247 (49.1)	0.447–0.535
Yes	256 (50.9)	0.465–0.553
**Hospitalization days (*n* = 256)**		
1–3	157 (61.3)	0.270–0.351
4–8	70 (27.3)	0.109–0.169
≥9	29 (11.3)	0.037–0.078
**Surgery**		
No	496 (98.6)	0.976–0.996
Yes	7 (1.4)	0.004–0.024
**Number of treatments**		
1	120 (23.9)	0.201–0.276
2	251 (49.9)	0.455–0.543
3	101 (20.1)	0.166–0.236
≥4	31 (6.1)	0.041–0.083

CI, Confidence interval. * Others include those animals electrocuted or poisoned.

Table 3 displays the multivariate analysis carried out to identify those variables associated with euthanasia in exotic animals. Non-hospitalized, birds, emergency visits, and the existence of musculoskeletal disorders had a significant impact on euthanasia. In addition, there was a significant year-on-year increase of 2.1 (CI: 1.1–3.8; *p* = 0.019).

**Table 3:** Multivariate ordinal logistic regression analysis of factors relevant to being euthanised.

Variables	OR (95% CI)	*p*-value
Hospital visit (emergency)	4.615 (1.005–21.199)	0.049
Animal class (birds)	6.811 (1.108–41.871)	0.038
System affected (musculoskeletal)	31.72 (4.184–240.4)	0.001
Hospitalization	2510.2 (206.4–30529.9)	<0.001
Year	2.078 (1.126–3.838)	0.019

CI, Confidence interval; OR, Odds ratio.

With respect to the treatments followed (*n* = 1,081), 1,069 (98.9%) were medications included in the ATCvet classification. Table 4 lists these treatments according to the first level of this classification, showing that nearly half of them belonged to group QN (Nervous system). When the fourth level was considered, the most commonly chemical/therapeutic subgroup was QB05BB (solutions affecting the electrolyte balance), which was used in 188 animals, followed by halogenated hydrocarbons (QN01AB, *n* = 186), oxicams (QM01AC, *n* = 173), and barbiturates (QN51AA) for animal euthanasia (*n* = 171). Specifically for each drug (fifth-level code), pentobarbital was administered to 187 animals (171 for animal euthanasia and 16 for sedation) followed by isoflurane (*n* = 184), meloxicam (*n* = 173), and electrolyte solutions (*n* = 153). It should be highlighted that 171 animals were euthanised with drugs from group QN (Nervous system). The HVULE protocol for birds consisted of inhaled isoflurane (5%) and intracardiac pentobarbital (0.1 g/kg). For mammals, intramuscular dexmedetomidine (0.04 mg/kg) or acepromazine (0.1 mg/kg), intravenous propofol (3 mg/kg) and intravenous pentobarbital (2 mg/kg) were used with the same purpose.

**Table 4:** Anatomical groups (first-level ATCvet) prescribed among exotic animals.

ATCvet anatomical group	Frequency (%) (*n* = 1,069)	95% CI
Group QA Alimentary tract and metabolism	10 (0.9)	0.004–0.015
Group QB Blood and blood-forming organs	196 (18.4)	0.160–0.207
Group QC Cardiovascular system	2 (0.2)	0.000–0.004
Group QD Dermatologicals	8 (0.7)	0.002–0.013
Group QG Genito urinary system and sex hormones	2 (0.2)	0.000–0.004
Group QH Systemic hormonal preparations, excluding sex hormones and insulins	13 (1.2)	0.006–0.019
Group QJ Antiinfectives for systemic use	133 (12.5)	0.105–0.144
Group QM Musculoskeletal system	176 (16.5)	0.142–0.187
Group QN Nervous system	497 (46.5)	0.435–0.495
Group QP Antiparasitic products, insecticides, and repellents	17 (1.6)	0.008–0.023
Group QS Sensory organs	4 (0.4)	0.000–0.007
**ATC anatomical group**		
Group S Sensory organs	11 (1.0)	0.004–0.016

CI, Confidence interval.

In group QN (Nervous system), subgroup analgesics (QN02) were also used (*n* = 111; 22.3%). In this case, all were opioids (QN02A), with a PD of 0.67. Parenteral buprenorphine (45.9%) was the main opioid employed (76.5% in birds and 23.5% in mammals), followed by tramadol (27.9%), butorphanol (22.5%), and methadone (3.6%). Butorphanol and methadone were only administered parenterally to birds and mammals, respectively, whereas tramadol was administered to both birds (90.3%) and mammals (9.7%) by parenteral (77.4%) or oral (22.6%) routes.

All prescriptions of the group QM (Musculoskeletal system) were non-steroidal anti-inflammatory drugs (NSAID), specifically meloxicam (*n* = 173) and robenacoxib (*n* = 3), with a PD for NSAID very small (0.03). Meloxicam was more commonly administered parenterally (79.2%) than orally (20.8%) and more frequently used in birds (85.5%) than mammals (14.5%). As for robenacoxib, this drug was administered only in mammals and by the parenteral route.

Group QJ01 *Antibacterials for systemic use* were used only in 12.5% of treatments, with a PD of 0.58. The most prescribed therapeutic subgroup was QJ01M *Quinolone and quinoxaline antibacterials* (Table 5), with marbofloxacin as the most used compound, followed by metronidazole.

**Table 5:** J01 therapeutic subgroups (third-level group ATCvet) prescribed among exotic animals.

ATCvet therapeutic subgroup J01	Frequency (%) (*n* = 133)	95% CI
Subgroup Q01C Beta-lactam antibacterials, penicillins	3 (2.3)	0.000–0.048
Subgroup Q01D Other beta-lactam antibacterials	4 (3.0)	0.001–0.059
Subgroup Q01E Sulfonamides and trimethoprim	13 (9.8)	0.047–0.148
Subgroup Q01F Macrolides, lincosamides and streptogramins	3 (2.3)	0.000–0.048
Subgroup Q01M Quinolone and quinoxaline antibacterials	94 (70.7)	0.638–0.791
Subgroup Q01X Other antibacterials	16 (12.0)	0.065–0.176

CI, Confidence interval.

**Figure 1:**
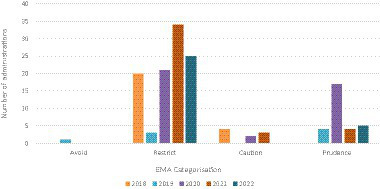
Antibiotic prescription according to the European Medicines Agency (EMA) categorization and year of study.

According to the EMA Categorization of antibiotics for prudent and responsible use in animals (38), only 1 (0.7%) of the active ingredients administered were classified as Avoid; 103 (72.0%) as Restrict; 9 (6.3%) as Caution; and 30 (21.0%) as Prudence. Most antibiotics (*n* = 90; 62.9%) were administered as parenteral treatments, followed by oral (*n* = 43; 30.1%) and local treatments (*n* = 10; 7.0%). The PD for all antibiotics was 0.63. Figure 1 shows the antibiotic prescriptions according to the EMA categorization and year of study. Altogether, 2.7 grams of antibacterials for systemic use was documented for exotic animals over the 5-year period (Table 6), with clear differences between birds and mammals. The quinolone and quinoxaline subgroup was the most prescribed one in both animal classes.

**Table 6:** Amount of QJ01 Antibacterials for systemic use; QM01A Antiinflammatory and antirheumatic products, non-steroids, and QN02A Opioids prescribed in exotic animals.

	Birds amount in grams	Mammals amount in grams	Total amounts in grams (%)
**QJ01 Antibacterials for systemic use**			
Subgroup Q01C Beta-lactam antibacterials, penicillins			0.0936 (3.5)
Amoxicillin	–	0.0089	0.0089 (0.3)
Amoxicillin and beta-lactamase inhibitor	–	0.0029	0.0029 (0.1)
Piperacillin and beta-lactamase inhibitor	–	–	0.0820 (3.0)*
Subgroup Q01D Other beta-lactam antibacterials			0.1368 (5.0)
Cefovecin	–	0.1398	0.1368 (5.0)
Subgroup Q01E Sulfonamides and trimethoprim			0.3505 (12.9)
Trimethoprim	–	0.1054	0.1054 (3.9)
Sulfamethoxazole and trimethoprim	0.1980	0.0472	0.2452 (9.0)
Subgroup Q01F Macrolides, lincosamides, and streptogramins			0.0606 (2.2)
Tilmicosin	–	0.0145	0.0145 (0.5)
Clindamycin	0.0461	–	0.0461 (1.7)
Subgroup Q01M Quinolone and quinoxaline antibacterials			1.4679 (54.2)
Enrofloxacin	–	0.1169	0.1169 (4.3)
Marbofloxacin	1.2361	0.1147	1.3509 (49.8)
Subgroup Q01X Other antibacterials			0.6007 (22.2)
Metronidazole	0.4296	0.1711	0.6007 (22.2)
Total	1.9098	0.7181	2.7100 (100)
**QM01A Antiinflammatory and antirheumatic products, non-steroids**			
Meloxicam	0.1092	0.0081	0.1173 (96.6)
Celecoxib	–	0.0041	0.0041 (3.4)
Total	0.1092	0.0122	0.1214 (100)
**QN02A Opioids**			
Buprenorphine	0.4298	0.0188	0.4486 (61.5)
Tramadol	0.2535	0.0206	0.2741 (37.5)
Methadone	–	0.0052	0.0052 (0.7)
Butorphanol	0.0021	–	0.0021 (0.3)
Total	0.6854	0.0446	0.7300 (100)

* Prescribed to a chelonian.

Table 7 summarizes the results of the multivariate analysis, identifying those variables associated with antibiotic prescription patterns in exotic animals. The need for hospitalization, being a mammal, non-emergency visits, and any condition different from digestive or musculoskeletal disorders had a significant impact on antibiotic prescription.

**Table 7:** Multivariate ordinal logistic regression analysis of factors relevant to the antibiotic prescription pattern.

Variables	OR (95% CI)	*p*-value
Hospital visit (no emergency)	2.190 (1.469–3.261)	<0.001
Animal class (mammals)	2.075 (1.385–3.109)	<0.001
Organ affected (Others vs. musculoskeletal)	2.081 (1.379–3.135)	<0.001
Organ affected (Others vs. digestive)	2.452 (1.462–4.115)	0.001
Hospitalization	2.477 (1.642–3.732)	<0.001
Year	1.121 (0.969–1.310)	0.121

CI, Confidence interval; OR, Odds ratio.

Furthermore, when comparing treatments with and without antibiotics, the likelihood of an antibiotic being administered parenterally was 1.8 times higher (OR = 1.8; 95% CI: 0.9–3.7; *p* = 0.119) than topically. The same happened for the oral route, which was 5.2 times higher (OR = 5.2; 95% CI: 2.4–11.5; *p* < 0.001) to be used than the topical one. Finally, if only the two main routes of administration were considered (oral and parenteral), antibiotics were 2.9 times more likely to be administered by the oral route than parenterally (OR = 2.9; 95% CI: 1.9–4.6; *p* < 0.001).

## 4 Discussion

To our knowledge, this is the first study which describes the pattern prescription in exotic pets and wildlife animals in a Veterinary Teaching Hospital over a long period of study. Most of the studies found in the literature are surveys (36, 41), and others are focussed on all types of pets (dogs, cats, and exotics) (40, 42).

As shown before, birds were the largest group of exotic animals attending the hospital over the study period. In Europe, 15.7% of pets are birds; 9.5%, small mammals; and 3.7%, reptiles. Italy, Turkey, and Spain have the largest populations of ornamental birds; Russia, Germany, and France of small mammals and France and Spain of reptiles (5). In Spanish households, the dog is the preferred pet (21.9%) followed by the cat (8.2%). However, the presence of other pets, mainly birds and fish, has increased considerably in the last years (43). In addition, according to a survey conducted at the University of Dublin with exotic pet veterinarians, birds occupied the largest number of their visits (96.4%), followed by small mammals (89.3%) and reptiles (78.6%) (44). Dogs and cats visited the veterinary at least once a year (45, 46). In contrast, only 50% of exotic pet owners said that they have visited a veterinary clinic in 2019 (47).

We have observed that birds went to the hospital for an emergency much more than small mammals. According to veterinary professionals, the percentage of emergencies seen daily is much higher in exotic animals than in dogs and cats (2). Several reasons may explain these differences. In this sense, these species may mask their illness until it is well advanced, and probably owners do not spend as much time observing their pets. In addition, they have specific environmental requirements, and their behavioral patterns of pain or illness are not still well understood (2).

Related to hospitalization, it should be taken into account that exotic animals usually visit the veterinarian when pathologies are much more advanced than in other domestic animals. This may be related to the lack of experience with these species, the equipment, or diagnostic tests specifically addressed to other animal species. In addition, the economic constraints of owners (44) for treatment or the lack of authorized medicines for these animals should be considered. In these situations, with very advanced pathological disorders and unfavorable prognosis, euthanasia must be done to reduce the animal’s suffering (48). It is important to perform this procedure using techniques that minimize stress (48). For this purpose, in 2013 the AVMA published guidelines for the euthanasia of exotic species (48). Acceptable methods of euthanasia include intravenous administration if performed without causing fear or distress. They recommend intramuscular deep sedation or prior anesthesia in these species, as carried out in the HVULE. Some drugs included in the AVMA guideline are xylazine, opioid analgesics, dexmedetomidine (used for small mammals in the HVULE), alfaxalone, or midazolam, among others (48). Once the animal is unconscious, euthanasia solutions are administered intravenously (route of administration used in small mammals in the HVULE), intraosseous, intracardiac, or intrathoracic (the latter two used in birds in this hospital) (48). Euthanasia rates were relatively higher (34.0%) compared to companion animals, such as dogs (1%), cats (2%), or rabbits (4%) (43), but more similar to values observed in backyard poultry (29.9%) (49). These authors related their euthanasia rates to the increased severity of the disorders in these animals (49).

Regarding pharmacological treatments, the most frequent group used was QN, with pentobarbital and isoflurane as the most often prescribed active ingredients, as one-third of the animals assessed in this study were euthanised. Opioids were also used quite frequently. Veterinarians prescribe and administer an important amount of opioid analgesics to treat pain in animals (50). Butorphanol is a synthetic opioid indicated for the treatment of acute pain in birds and small mammals (51, 52), recommended in birds as preoperative and postoperative analgesic medication (51–56), and for mild pain and short-term analgesia in small mammals (57, 58). As for buprenorphine, it is commonly administered in veterinary medicine for analgesia and sedation, although little information is available on its use in exotic animals. It is not recommended for pain control in reptiles, but used in birds, whereas in small mammals there is more research providing evidence of its analgesic and sedative effects (59).

The second most used group was QB (blood and blood-forming organs), in certain solutions affecting the electrolyte balance, which is in accordance with the fact that half of our patients were hospitalized and received fluid therapy. Fluid administration is essential to treat many medical conditions or provide support therapy to patients. Regarding routes of administration, subcutaneous fluids are a valid treatment of choice for hypovolemic avian and reptile patients. In particular, electrolyte solutions are an option for the majority of exotic animal fluid treatment needs (60).

Within the QM group, meloxicam was used quite often as a high number of patients had musculoskeletal disorders. This drug is commonly prescribed off-label for pain and inflammation in many exotic and zoo animals, including reptiles and birds (61). Nowadays, it is the current drug of choice due to its widespread use and low incidence of reported toxicity in exotic animal practices. In a study in which this drug was administered to over 700 captive birds (60 different species) in zoos, no mortality was observed (62). In addition, several studies reported no significant renal, gastrointestinal, or haemostatic adverse events at the doses evaluated (63–66). Vultures are more sensitive to the renal adverse effects of several NSAIDs, except for meloxicam (67). The preference for this NSAID is also reflected in the low PD of our veterinary hospital for the population assessed.

Inappropriate use of antimicrobials is a potential threat to public health (34). Multidrug-resistant bacteria have been reported in several exotic animal species, such as methicillin-resistant staphylococci in rabbits and birds, extended-spectrum β-lactamase-producing enterobacteria in turtles and wild birds, or resistant *Escherichia coli* in wild birds (28, 68–72). Specifically in the Iberian Peninsula, *Pseudomonas* spp. showed the highest levels of resistance among birds, mammals, and reptiles, and multidrug resistance was also significant in *Enterobacterales* (73). In 2015, WHO adopted a global action plan to address antimicrobial resistance and proposed to ban the prophylactic use of antimicrobials in healthy animals (74). To this end, the EU has significantly restricted the prophylactic use of antimicrobials (32). Moreover, since 2014, the EMA has categorized antibiotics for veterinary use taking into account the public health risk of their use in animals due to the potential development of resistance, as well as the need for their use (38).

For those antibiotics belonging to category B (Restrict), we found that marbofloxacin was the most widely used in the HVULE. Fluoroquinolones are frequently used in birds, mainly due to their wide safety margin. However, injectable solutions are highly irritant and may cause tissue necrosis (75). In addition, some studies have recognized that fluoroquinolones have detrimental effects on reproduction in scavenging birds (76). In general, these antibiotics that reduce hatchability and total egg hatch, and cause joint deformities in chicks, increased embryonic heart rate and biochemical signs of stress (77). Since the 1980s, the livestock industry has been using fluoroquinolones as antimicrobials in food-producing animals (78, 79). A 1998–2001 survey of UK veterinarians on dermatological treatments showed that the most commonly used antibiotics in dogs, cats, and exotic animals were cefalexin, amoxicillin, and enrofloxacin (41). In another study carried out in the UK in 2016, fluoroquinolones were the most usually prescribed antibiotic group (49%) in rabbits and third-generation cephalosporins (36%) in cats and amoxicillin with clavulanic acid (29%) in dogs (40). In Germany, amoxicillin with clavulanic acid was the most prescribed drug in dogs (47.89%) and cats (48.15%) in 2017–18 (42). In contrast, in backyard poultry, the most prescribed antibiotic was enrofloxacin (40.6%), followed by tylosin (19.5%) and amoxicillin with clavulanic acid (12.1%). Furthermore, 92.4% of those antibiotics prescribed for chickens were for systemic administration (oral or injectable) and only 5.2% for topical administration (49). Another survey among Swiss veterinarians on antibiotics usage in exotic pets showed that fluoroquinolones were the most commonly prescribed drugs in rabbits (82%), rodents (86%), birds (83%), and reptiles (97%) (36).

We have observed that antibiotics from category A (Avoid), which should be given to companion animals under exceptional circumstances, were used only once at the HVULE, whereas those belonging to category B (Restrict), whose use should be restricted in animals due to their critical importance in human medicine, were prescribed in the hospital quite frequently. However, antibiotics belonging to category C (Caution), which should be used only when there are no effective category D (Prudence) antimicrobials, are minimal. Finally, although category D is the first group of choice, their use is not very high in our study. The prescription of antimicrobials classified in EMA categories A and B should be based on antimicrobial susceptibility testing whenever possible (38). Some countries, such as the Netherlands (80), Belgium (81), and Denmark (82), have national legislation that requires susceptibility testing to verify that no other antibiotics in categories C or D are clinically effective before an antimicrobial belonging to fluoroquinolones or third−/fourth-generation cephalosporins can be prescribed, and it is also the case in Spain (33).

As for the PD of antibiotics, our value was 0.63, which is much lower than those reported in 2016 for dogs (0.83) and cats (0.75), and very similar to that determined for rabbits (0.64) in the UK (40). In 2018, the prevalence of PD data increased in Germany for dogs and cats to 0.93 and 0.88, respectively (42). Our data may reflect that evidence-based guidelines for the treatment of infections, including antimicrobial stewardship, are not yet available for exotic animals. In the absence of appropriate veterinary medicines, common practice is based on personal experience of successful strategies implemented by individual veterinarians. As a result, off-label use of critical antimicrobials such as fluoroquinolones, aminopenicillins, third-generation cephalosporins, or macrolides is frequent in exotic pets, which underscores the need for guidance for prudent antibiotic use. The positive influence of these recommendations has already been demonstrated in dogs and cats (34).

Potential limitations of this study include the representativeness of the sample, and results may not be generalisable to other clinics or veterinary hospitals. Although significant efforts were made to identify animal species, demographic characteristics, clinical signs, and pharmaceutical prescriptions using manual and semi-automated methodologies, unclear or missing descriptions may have been overlooked by the authors. Additionally, the COVID-19 pandemic occurred during the period of study and may have had an impact on hospital visits. However, despite these limitations, our findings may help raise further discussions on the prudent use of several pharmacological groups in these types of animals, which are becoming more and more popular among the population.

This is the first study that describes the actual consumption of drugs in exotic pets and wild animals at a Veterinary Teaching Hospital. We have observed that the number of exotic animals increased over time, and they were mainly birds who went to the hospital for an emergency related to the QM group, and one-third were euthanised. As for medicines, almost half belonged to the QN group, with pentobarbital as the most used drug, followed by isoflurane and meloxicam. Regarding antibiotics, marbofloxacin (Category B—Restrict) was the most prescribed one.

Our study provides an insight into the prescription patterns in exotic animals. The findings of the study provide sufficient data to veterinary policymakers and education aimed at improving drug use practices in general and antimicrobial use, in particular in the profession.

## Data availability statement

The raw data supporting the conclusions of this article will be made available by the authors, without undue reservation.

## Ethics statement

Data used in this study are based on these generated for accounting and documentation purposes. Our research does not involve any regulated animals, and no scientific procedures of any kind were performed on animals. For this reason, formal approval by an ethics committee was not necessary under the provisions of the Spanish regulations.

## Author contributions

BR: Conceptualization, Methodology, Writing – original draft, Writing – review & editing. JS: Formal Analysis, Writing – review & editing. AS: Data curation, Writing – review & editing. NF: Data curation, Writing – review & editing. CL: Project administration, Writing – review & editing. RP: Resources, Writing – review & editing. JA: Writing – review & editing. RD: Conceptualization, Methodology, Project administration, Writing – review & editing.
